# Molecular Mechanism of Interaction between DNA Aptamer and Receptor-Binding Domain of Severe Acute Respiratory Syndrome Coronavirus 2 Variants Revealed by Steered Molecular Dynamics Simulations

**DOI:** 10.3390/molecules29102215

**Published:** 2024-05-09

**Authors:** Xuan Ding, Chao Xu, Bin Zheng, Hanyang Yu, Peng Zheng

**Affiliations:** 1 Department of Biomedical Engineering, College of Engineering and Applied Sciences, Nanjing University, Nanjing 210093, China; 2 State Key Laboratory of Analytical Chemistry for Life Science, Nanjing University, Nanjing 210093, China; 3 School of Chemistry and Chemical Engineering, Nanjing University, Nanjing 210093, China

**Keywords:** DNA aptamer, SARS-CoV-2, SMD simulations

## Abstract

The ongoing SARS-CoV-2 pandemic has underscored the urgent need for versatile and rapidly deployable antiviral strategies. While vaccines have been pivotal in controlling the spread of the virus, the emergence of new variants continues to pose significant challenges to global health. Here, our study focuses on a novel approach to antiviral therapy using DNA aptamers, short oligonucleotides with high specificity and affinity for their targets, as potential inhibitors against the spike protein of SARS-CoV-2 variants Omicron and JN.1. Our research utilizes steered molecular dynamics (SMD) simulations to elucidate the binding mechanisms of a specifically designed DNA aptamer, AM032-4, to the receptor-binding domain (RBD) of the aforementioned variants. The simulations reveal detailed molecular insights into the aptamer–RBD interaction, demonstrating the aptamer’s potential to maintain effective binding in the face of rapid viral evolution. Our work not only demonstrates the dynamic interaction between aptamer–RBD for possible antiviral therapy but also introduces a computational method to study aptamer–protein interactions.

## 1. Introduction

The global pandemic caused by Severe Acute Respiratory Syndrome Coronavirus 2 (SARS-CoV-2) has caused unprecedented devastation on human society. This pathogen’s high virulence, infectivity, and rapid mutation rate have posed significant challenges to global health systems [1,2,3]. SARS-CoV-2 has infected nearly one billion human beings and caused about seven million deaths so far. In an effort to combat this deadly virus, scientists and researchers worldwide have rallied to develop several vaccines targeting the spike protein of SARS-CoV-2 [4]. These vaccines have played a pivotal role in preventing the spread of the disease and, in many cases, reducing the severity of the symptoms experienced by infected individuals [5]. Despite the vaccines’ critical role in controlling the outbreak, their deployment has been accompanied by concerns over potential adverse effects associated with antibody-based therapies. Notably, the phenomenon of Antibody-Dependent Enhancement (ADE) poses a risk that may exacerbate disease severity under certain conditions, underscoring the complexity of the immune response to viral infections and the need for a nuanced understanding of vaccine-induced immunity.

**Figure 1:**
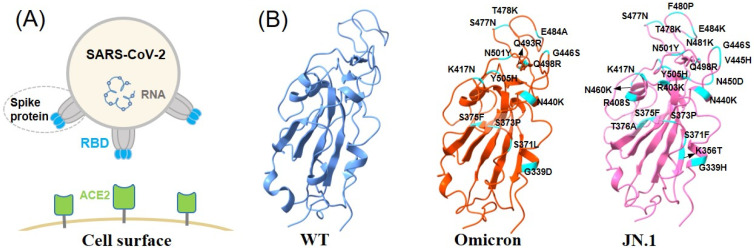
(**A**) SARS-CoV-2 attaches to epithelial cells on the surface via its spike protein’s receptor-binding domain (RBD, colored in blue), interacting with ACE2 (colored in green). (**B**) The structure of three RBDs are shown, respectively. From left to right is the wild-type, Omicron, and JN.1. The residues colored in cyan are the mutations compared to wild-type RBD.

Furthermore, the rapid evolution of SARS-CoV-2, evidenced by the emergence of variants such as Alpha, Beta, Omicron, and the very recent JN.1 variant (Figure 1), presents a major challenge for antibody/vaccine treatment [6,7,8]. As the primary protein of the virus that binds to the angiotensin-converting enzyme 2 (ACE2) receptor of the host cell, the spike protein plays an essential role in the infectivity and transmissibility of the virus, showing an exceptionally high evolutionary rate. The receptor-binding domain (RBD) of the spike protein is the major adhesion protein for viral entry which binds specifically to ACE2 as its receptor. These variants have shown many mutations on the RBD of the spike protein and thus have demonstrated the ability to partially escape the immune response elicited by vaccines and antiviral drugs, thereby reducing the overall efficacy of these preventive measures. 

The Omicron variant, for example, has presented with over 30 mutations in the spike protein, with a significant concentration in the RBD. This has implications for vaccine efficacy and highlights the importance of continuous monitoring and analysis of emerging variants. Similarly, the JN.1 variant, which emerged in late 2023, carries mutations that have been linked to increased transmissibility and an enhanced ability to evade immune responses. It harbors Leu455Ser and three mutations in non-spike proteins [9]. Consequently, there is a need for ongoing research and the development of alternative therapeutic strategies to address the potential for more severe future SARS-CoV-2 variants.

In this context, the search for alternative therapeutic strategies that can offer broad-spectrum efficacy against current and future SARS-CoV-2 variants is more critical than ever. One of the most promising of these alternative strategies is the use of aptamers [10,11]. Aptamers are short, single-stranded oligonucleotides (either DNA or RNA) known for their high affinity and specificity for various target molecules, including proteins [12,13,14]. Unlike neutralizing antibodies, aptamers offer several distinct advantages in the context of SARS-CoV-2 treatment. They can be chemically synthesized from nucleotides at a relatively low cost and are amenable to rapid modifications. Additionally, the inherent thermal stability of aptamers, derived from their nucleic acid composition, allows them to be stored and transported at room temperature without degradation over extended periods. The relatively small size of aptamers facilitates their delivery into the respiratory system through intranasal administration or nebulization, making them particularly suitable for treating respiratory viruses like SARS-CoV-2. Importantly, unlike antibodies which contain an Fc fragment, aptamers are less susceptible to triggering ADE, potentially making them safer for a broader range of applications across different species.

Recent scientific endeavors have led to the development of aptamers that specifically target the RBD of SARS-CoV-2 and its variants, laying the groundwork for their use in both diagnostic and therapeutic settings [15]. A notable advancement in this area is the creation of an DNA aptamer named AM032-0 through the Systematic Evolution of Ligands by Exponential Enrichment (SELEX) technique [16,17]. It has demonstrated strong binding affinity to the spike proteins of various SARS-CoV-2 variants, within the nanomolar (nM) range. 

**Figure 2:**
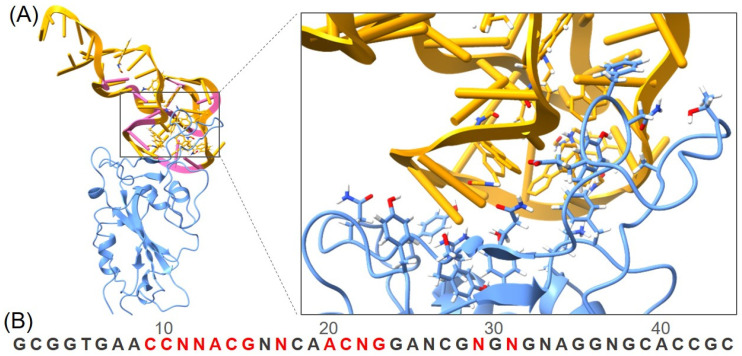
(**A**) The structure of the RBD-DNA aptamer complex (RBD in blue, DNA aptamer in yellow and the modified nucleotide in pink), along with a focused image of the binding interface. DNA aptamers can target and bind to the RBD, thereby hindering RBD-ACE2 interaction. (**B**) Sequence of the DNA aptamer, with the bases interacting with the RBD colored in red. N, Nap-dU = 5-[N-(1-naphthylmethyl) carboxamide]-2′-deoxyuridine.

This particular aptamer is composed of approximately 80 nucleotides, including 11 modified Nap-dU nucleotides that confer unique structural properties, such as a three-turn structure stabilized by a hydrophobic core. This core is primarily composed of seven Nap-dU residues, with the first turn (T1) playing a critical role in binding to the RBD of the virus as demonstrated by the complex structure obtained from cryo-EM. Moreover, the original aptamer AM032-0 is further optimized for increased binding affinity and shorter length. Accordingly, a much shorter 44 base-length DNA aptamer AM032-4 is obtained, showing strong binding affinity to the spike proteins at the nM level (Figure 2). 

As the battle against COVID-19 continues, especially with the virus changing so rapidly, aptamers could play a key role in developing new treatments. Their adaptability, ease of use, and effectiveness against the virus’s spike protein make them a powerful tool in our arsenal against current and future coronavirus threats. In this work, we use an advanced computational method to study how exactly these DNA aptamers work against the virus to uncover their interactions with the virus’s variants.

## 2. Result and Discussion

To further investigate the binding mechanism of the DNA aptamer to the RBD of SARS-CoV-2 variants, especially to assess its potential efficacy against newer variants such as JN.1, we employed steered molecular dynamics (SMD) simulations to study the complex unbinding process. SMD simulations are a powerful tool for visualizing the dissociation process of receptor–ligand complexes under applied mechanical force, providing deep molecular insights into the interaction [18,19]. This approach offers unparalleled molecular insights, shedding light on the potential efficacy of AM032-4 against these evolving threats.

**Figure 3:**
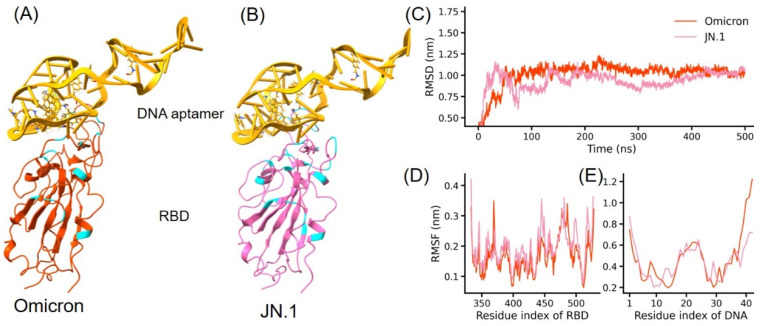
(**A**,**B**) represent the complex structures of the DNA aptamer with the RBDs of the SARS-CoV-2 Omicron variant and the JN.1 variant, respectively. Cyan denotes residues at positions where mutations have occurred relative to the wild-type RBD. (**C**–**E**) represent the RMSD results of the RBD-DNA aptamer during a 500 ns MD simulation. The orange and pink lines in (**C**) represent the RMSD analysis of the Omicron RBD-DNA complex and the JN.1 RBD-DNA complex, respectively. (**D**,**E**) depict the RMSF analyses of the RBD and DNA, respectively, over the 500 ns trajectory.

**Figure 4:**
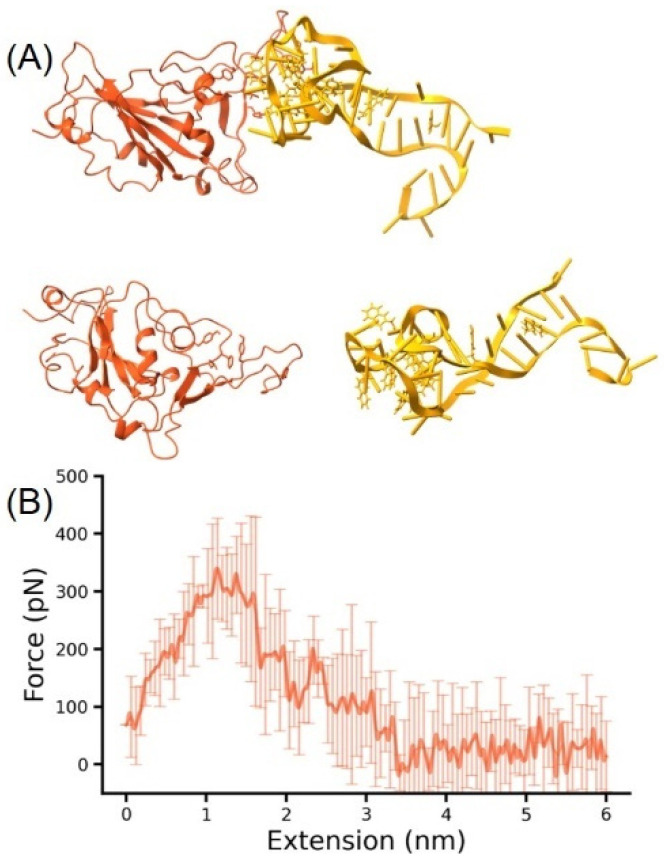
(**A**) A schematic of the steered molecular dynamics simulations for the Omicron RBD-DNA aptamer, with the RBD fixed and the DNA aptamer stretched by a harmonic force (**top**), leading to complex dissociation (**bottom**). (**B**) A graph of the force generated during the SMD process as a function of distance, where the solid line represents the average of multiple results, and the transparent lines represent the standard deviation at that point.

We first built the structure of Omicron RBD-DNA aptamer AM032-4 complex (Figure 3). A close binding between the two molecules was observed, highlighting their direct interactions pivotal for viral inhibition. In the SMD simulations, the RBD was fixed and the aptamer was pulled. As the aptamer was pulled away, the force gradually increased. Notably, the complex involving Omicron exhibited the highest unbinding force at an extension of 1.0 nm, reaching up to 350 pN (Figure 4). This aligns with the existing literature, confirming AM032-4′s potent affinity for the Omicron variant’s RBD. The vivid depiction of the unbinding process—illustrating the strong interaction before culminating in the final separation—underscores the aptamer’s mechanical stability and therapeutic potential. It shows the strong interaction between the two molecules. Then, the force decreased and they separated at the extension around 3 nm. This phenomenon is crucial for understanding the mechanical stability and efficacy of aptamer-based therapeutic interventions against SARS-CoV-2. 

A noteworthy observation was the DNA aptamer’s transition from a compact to a classic double-stranded form post-dissociation. This suggests an RBD-binding-induced conformational shift in AM032-4. It may enhance its affinity for the RBD. Such findings show the importance of mechanical force and stability in the RBD-ACE2 receptor interaction, highlighting SMD simulations’ role in bridging experimental studies. Nevertheless, another explanation is possible. For example, during stretching, the presence of foreign body hydrogen bonding may stabilize DNA in this particular conformation The conformational transition observed in AM032-4 reveals the dynamic nature of aptamer–RBD interactions. This RBD-binding-induced conformational change likely contributes to an increased binding affinity, representing a critical mechanism by which aptamers exert their inhibitory effects. Understanding these conformational dynamics is crucial for the design of next-generation aptamers with optimized therapeutic properties. Indeed, previous studies have emphasized the importance of mechanical force and stability in the binding interaction between the RBD and ACE2 receptor, as well as in the evolution of the RBD, underscoring the value of SMD simulations in complementing experimental findings.

**Figure 5:**
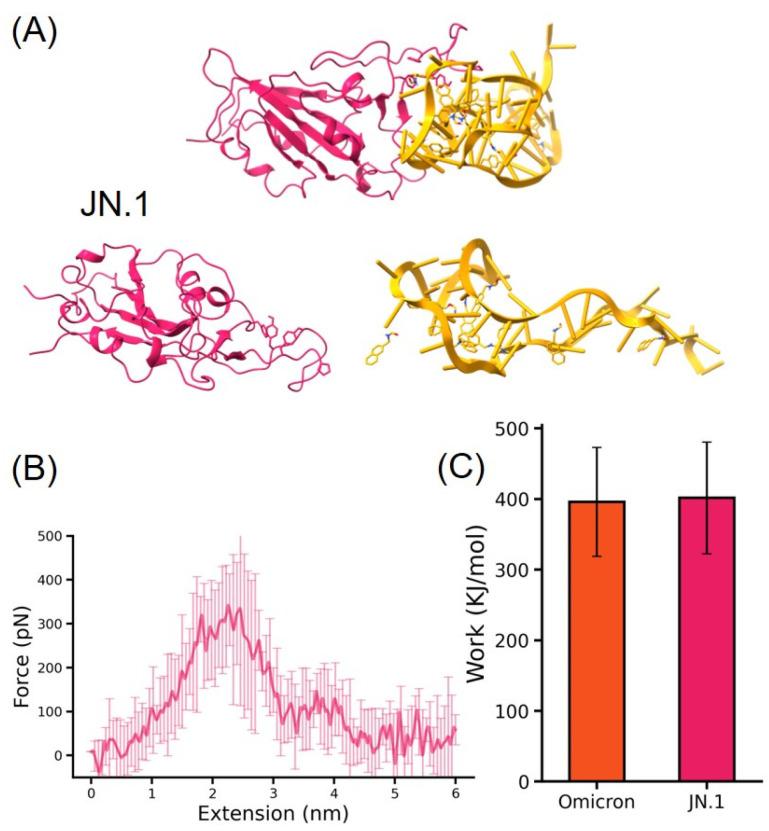
(**A**) A schematic of the steered molecular dynamics simulations for the JN.1 RBD-DNA aptamer, with the same setup as that for Omicron, where RBD is fixed and the DNA aptamer is stretched by a harmonic force. (**B**) A graph of the force generated during the SMD process as a function of distance. A force peak with maximum force of 350 pN is observed at an extension around 2.5 nm. (**C**) In the process of SMD simulation, the work required to separate the RBD and DNA aptamer by an external force for Omicron and JN.1, respectively.

Next, we built the structure of JN.1 RBD-DNA aptamer AM032-4 complexes for SMD simulations. For JN.1, a longer extension of 2 nm was observed for reaching the maximum force during SMD simulation (Figure 5), and the interaction gradually disappeared at the extension of 3 nm. The JN.1 complex demonstrated an unbinding force of 350 pN, which is similar to Omicron. Thus, this high force value indicates the potential of AM032-4 to also bind to the newly emerged JN.1 variant. In addition, a longer extension showed the difference between JN.1 and Omicron. A more and stronger interaction is possible for JN.1 as well. The unbinding forces observed in our study are not only consistent with AM032-4′s known binding efficacy but also provide a comparative lens through which to view aptamer interactions across variants. The similar unbinding forces for Omicron and JN.1—despite the latter’s extended dissociation pathway—suggest a broadly effective binding mechanism that transcends specific mutations within the RBD. This implies that AM032-4 and potentially other aptamers could serve as versatile tools in the antiviral arsenal against current and emerging SARS-CoV-2 variants. 

Our application of steered molecular dynamics (SMD) simulations to dissect the interactions between the DNA aptamer AM032-4 and the RBD of SARS-CoV-2 variants, specifically Omicron and JN.1, heralds a pivotal advancement in antiviral strategy development. These simulations offer a window into the atomic-level dynamics of aptamer–protein interactions, enabling us to visualize the step-by-step dissociation of the aptamer–RBD complexes under mechanical forces. This granular insight is invaluable for conceptualizing new therapeutic approaches that are both innovative and effective against a rapidly mutating virus. The structural integrity and interaction patterns of the Omicron RBD-DNA aptamer and JN.1 RBD-DNA aptamer complexes underscore the specificity and adaptability of AM032-4 to distinct viral variants. Observing a maximum unbinding force of 350 pN at a 1.0 nm extension for Omicron and a similar force at a 2.5 nm extension for JN.1 enriches our understanding of the physical underpinnings of aptamer efficacy. This differential extension requirement between Omicron and JN.1 complexes hints at variant-specific interaction nuances that could guide the refinement of aptamer designs for enhanced specificity and binding strength.

Molecular dynamics (MD) simulations provide a powerful method approach for studying molecular behavior and understanding their properties [20,21,22,23,24]. These simulations enable researchers to gain insights into various phenomena [19,25,26]. As a special type of MD, SMD simulations apply an external potential energy on biomolecules and mimic the mechanical (un)folding and (un)binding experiments performed by single-molecule force spectroscopy (SMFS) [27,28,29,30,31,32,33,34,35]. It can show the dynamic unfolding or unbinding trajectory for the protein or the complex, providing the critical molecular mechanism underlying the process. As a result, the key step or intermediate state can be revealed. In addition, the unfolding force or dissociation force can be measured in silico, providing a qualitative result for comparison. Thus, it is highly complementary to SMFS experiments and many proteins have been studied both by SMFS techniques and SMD simulations. 

On the other hand, SMFS techniques, such as atomic force microscopy (AFM), optical and magnetic tweezers, have been widely used to manipulate protein molecule(s) mechanically for the past several decades [36,37]. It can be used to studied the receptor–ligand interaction like the protein–aptamer interaction studied here. In particular, the interaction between the viral adhesion protein and its receptor have been intensively studied by SMFS [29,38]. For SARS-CoV-2, which may experience mechanical force during human sneezing and coughing, this technique mimics the external force from the dynamic airway on the protein during measurement. Thus, SMFS manipulation on the spike protein/RBD-ACE2 complex is physiologically relevant and has been studied [39]. For example, several groups have used SMFS to study the binding strength between the RBD and ACE2, especially for the various viral variants [40]. They found that SARS-CoV-2 shows a higher binding force and binding energy to ACE2 than SARS-CoV-1 [19,20]. We studied the effects of SARS-CoV-2 mutations and found that the N501Y mutation enhances RBD binding to ACE2, while K417N and E484K do not [21]. In addition to measuring protein–protein interaction, AFM-SMFS has been most widely used to measure the mechanical stability of the protein by protein (un)folding experiments, such as the giant muscle protein titin and many other mechanically stable proteins [41,42]. They showed an unfolding force of hundreds of piconewtons, which is a standard for mechanically stable protein. Additionally, AFM-SMFS has been used in the cellular level. Here, our SMD simulations result on the RBD–aptamer complex showed it is of ~300 pN. Although the pulling speed is different from the simulations and experiment, the clear force peak still suggests it can be a nice subject for further AFM-SMFS experiments and may show a detectable unbinding force [43,44,45,46,47,48,49]. We anticipate these studies of protein–DNA interaction in the near future. 

Coronaviruses are large, single-stranded RNA viruses evolving with a remarkable mutation rate, as evidenced by the transmission of several VOCs of SARS-CoV-2 in only the past three years. In addition to neutral mutations, the effect of many accumulated mutations has been revealed. Our application of SMD simulations has illuminated the unbinding process of the RBD-DNA aptamer complex, providing a detailed molecular perspective on the interaction between these two entities. The rapid mutation rate of coronaviruses, as highlighted by the swift emergence of several Variants of Concern (VOCs) over a short span, underscores the pressing need for adaptable and resilient therapeutic strategies. Our findings from the SMD simulations shed light on how specific mutations in the RBD affect the unbinding process and, by extension, the overall binding efficacy of therapeutic aptamers. By providing a molecular-scale understanding of these interactions, we contribute to a growing knowledge base that supports the development of targeted, mutation-resistant antiviral therapies. By showcasing the dissociation dynamics and quantifying the unbinding forces involved, our study contributes significantly to the growing body of knowledge on aptamer-based strategies for combating SARS-CoV-2 and its variants. The successful visualization and characterization of these processes underscore the critical role of computational methods, such as SMD simulations, in advancing our understanding of viral inhibition mechanisms and guiding the development of effective therapeutic interventions. 

The promising results from our investigation into the AM032-4 aptamer’s interaction with SARS-CoV-2 variants emphasize the potential of aptamers as versatile, effective antiviral agents. Their ability to be rapidly synthesized and tailored to specific targets offers a significant advantage over traditional antibody therapies, particularly in the face of a virus that continually evolves to evade immune detection and neutralization. Our study not only illuminates the molecular intricacies of aptamer–virus interactions but also paves the way for the broader application of computational simulations in virology and drug development. As we advance our understanding of the dynamic interplay between therapeutic aptamers and viral proteins, we open new avenues for the design of antiviral compounds capable of outpacing viral mutation rates. Future research will undoubtedly expand upon our findings, exploring the vast landscape of aptamer therapeutics with the aim of harnessing their full potential in the fight against not only SARS-CoV-2 but also other viral pathogens that threaten global health.

In conclusion, the integration of steered molecular dynamics simulations into the study of aptamer-based interventions against SARS-CoV-2 represents an important step forward in our quest to develop adaptable, effective antiviral strategies. To our best knowledge, using SMD simulations to study the dynamic interaction between protein and DNA aptamer is rare. By delving deep into the molecular mechanisms that underpin aptamer binding and inhibition, we lay a foundation for the next generation of antiviral therapies—ones that are precisely engineered to combat the virus at the molecular level, offering hope in the face of an ever-evolving virus.

## 3. Materials and Methods

### 3.1. Structure Modeling

Utilizing the crystal structure of the receptor-binding domain (RBD) in conjunction with a DNA aptamer (PDB code: 8J26) as a reference, Homology Modeling techniques were employed to predict the structures of the RBDs specific to the Omicron variant and the JN.1 variant. This modeling procedure was executed using the Swiss Model platform, which facilitates comparative protein structure modeling. The primary amino acid sequences corresponding to the Omicron and JN.1 RBDs were acquired from the Global Initiative on Sharing All Influenza Data (GISAID) database, a prominent repository for genomic data pertaining to various infectious diseases, including viral variants. After constructing the structure of RBDs, it was superimposed onto the wtRBD-DNA complex, followed by substitution of the mutant RBD (Omicron and JN.1) for wtRBD.

### 3.2. MD Simulation Force Field Preparation

Given the presence of additional chemical modifications within the DNA bases constituting the DNA aptamer, standard residues specified by the CHARMM36 force field are inadequate for accurately representing these modified nucleotides. Consequently, a tailored force field suitable for accommodating such non-natural residues was constructed through the utilization of the CGenFF methodology. This process involves the parameterization of force field parameters, including bond lengths, bond angles, dihedral angles, and van der Waals parameters, to ensure compatibility with the structural characteristics and chemical properties of the modified DNA bases.

Following the generation of the specialized force field, an essential aspect in the preparation of the molecular system for subsequent simulations involves the assignment of accurate partial charges to the constituent atoms. To achieve this, the Restrained ElectroStatic Potential (RESP) charges were calculated using computational methods implemented within AmberTools21 [50,51]. RESP charges are derived by fitting the electrostatic potential generated by the molecular system to a set of predefined reference charges, thereby ensuring that the resulting charge distribution accurately reflects the molecular structure and electronic environment of the system.

The process of RESP charge calculation involves several steps, including geometry optimization of the molecular system, computation of electrostatic potentials at predefined grid points surrounding the molecules, and subsequent fitting of these potentials to obtain the partial charges for each atom. This meticulous procedure ensures the generation of reliable charge parameters essential for conducting molecular dynamics simulations with high accuracy and predictive power.

### 3.3. MD Simulation for RBD-DNA Aptamer Unbinding

The investigation into the unbinding force dynamics of the RBD-DNA aptamer complex involved a series of methodical steps. Initially, steered molecular dynamics (SMD) simulations were chosen as the methodology, facilitated by the GROMACS software platform. The setup of simulation systems was meticulously undertaken using the CHARMM-GUI toolkit, ensuring precise arrangement and orientation of the molecular components. These systems were based on the structures of the RBD-DNA aptamer complexes as previously prepared. Utilizing the GROMACS 2023.3 package [52,53,54], the SMD simulations commenced, guided by the CHARMM36 force field complemented by the TIP3P water model. This combination allowed for accurate representation of intermolecular interactions within the simulation environment. To maintain consistency with physiological conditions, all simulations were conducted under periodic boundary conditions within the canonical NpT ensemble, with the temperature held constant at 298 K throughout the simulation duration [55].

Before initiating the molecular dynamics (MD) simulations, a rigorous energy minimization protocol was applied to all systems. This protocol, comprising 5000 steps, aimed to alleviate steric clashes and unfavorable interactions within the molecular structures. Following energy minimization, a phase of pre-equilibration was conducted, involving MD simulations with position restraints specifically on the protein backbone atoms. This pre-equilibration phase, lasting 125 picoseconds, was crucial for stabilizing the systems prior to the main MD runs, which extended over a duration of 500 ns. To ascertain the equilibration status of the system, subsequent RMSD and RMSF analyses were conducted. Specifically, initially, the periodic boundary conditions were removed from the simulations using gmx trjconv. Subsequently, the built-in program “rms” in GROMACS was employed to perform RMSD analysis on the RBD-DNA complex. Following this, the built-in program “rmsf” was utilized to analyze the fluctuation of residues within the complex.

To characterize the unbinding force dynamics of the RBD-DNA aptamer complex in detail, a series of five SMD simulations were conducted. Each SMD simulation employed a constant stretching velocity, with a pulling speed set at 1 nm/ns. Additionally, a harmonic constraint force of 1000 kJ/mol/nm^2^ was applied for a duration of 6.0 ns in each simulation. During this phase, the SMD methodology was employed by harmonically restraining the position of the RBD (either Omicron or JN.1) while exerting a pulling force on the DNA aptamer. This approach facilitated the controlled separation of the complex, allowing for precise measurement of the unbinding forces between the molecular components.

To comprehensively assess the energy changes during the SMD simulation process, a Python script was utilized to calculate the work done during each stretching event. Specifically, the force–distance curves generated during the SMD process were analyzed. The trapz function from the numpy library was employed to numerically integrate the area under the curve, thereby accumulating the work conducted throughout the entire SMD simulation process. The numerical calculations in this work have been performed on the computing facilities in the High-Performance Computing Center (HPCC) of Nanjing University. 

## Data Availability

All data is present in the manuscript.
